# Propagation Methods Decide Root Architecture of Chinese Fir: Evidence from Tissue Culturing, Rooted Cutting and Seed Germination

**DOI:** 10.3390/plants11192472

**Published:** 2022-09-21

**Authors:** Linxin Li, Xianhua Deng, Ting Zhang, Yunlong Tian, Xiangqing Ma, Pengfei Wu

**Affiliations:** 1 College of Forestry, Fujian Agriculture and Forestry University, Fuzhou 350002, China; 2 Chinese Fir Engineering Technology Research Center of the State Forestry and Grassland Administration, Fuzhou 350002, China

**Keywords:** *Cunninghamia lanceolata*, tree propagation methods, root system growth development, phenotypic characteristic, resource acquisition features, axial root

## Abstract

The root is the main organ of a plant for absorbing resources and whose spatial distribution characteristics play an important role in the survival of seedlings after afforestation. Chinese fir (*Cunninghamia lanceolata*) is one of China’s most important plantation species. To clarify the effects of propagation methods on root growth and spatial distribution characteristics of Chinese fir trees, sampled trees cultivated by seed germination, tissue culture, and asexual cutting of Chinese fir were taken as the research objects. The root morphology, geometric configuration, and spatial distribution characteristics of different trees were analyzed. The influence of geometric root morphology on its spatial distribution pattern was explored by correlation analysis, and the resource acquisition characteristics reflected by the roots of Chinese fir trees with different propagation methods are discussed. The main results showed that the root mean diameter (1.56 mm, 0.95 mm, and 0.97 mm), root volume (2.98 m^3^, 10.25 m^3^, and 4.07 m^3^), root tip count (397, 522, and 440), main root branch angle (61°, 50° and 32°) and other geometric configurations of Chinese fir under seed germination, tissue culture and rooted cutting respectively, were significantly different, which resulted in different distribution characteristics of roots in space. Chinese fir seed germination had noticeable axial roots, and the growth advantage was obvious in the vertical direction. A fishtail branch structure (*TI* = 0.87) was constructed. The shallow root distribution of tissue culture and rooted cutting was obvious, and belonged to the fork branch structure (*TI* = 0.71 and 0.74, respectively). There was a tradeoff in the spatial growth of the root system of Chinese fir trees with different propagation methods to absorb nutrients from heterogeneous soil patches. A negative correlation was present between the root system and root amplitude. There was an opposite spatial growth trend of Chinese fir trees with different propagation methods in the vertical or horizontal direction. In conclusion, selecting suitable propagation methods to cultivate Chinese fir trees is beneficial to root development and the “ideal” configuration formation of resource acquisition to improve the survival rate of Chinese fir afforestation.

## 1. Introduction

Plant offspring can be cultivated by sexual or asexual propagation, and the individual size, phenotypic traits, life history characteristics, genetic characteristics, and life strategies in the adversity of different propagation methods are different [1]. Sexual propagation can greatly maintain species’ genetic diversity, but gene recombination during propagation is not easy for maintaining the excellent characteristics of species. Asexual propagation methods such as tissue culture, cutting, grafting, and squashing can realize the rapid proliferation of seedlings and maintain the excellent characteristics of varieties. Using this relatively “low consumption” breeding cost to cultivate seedling afforestation has become an important way to expand the plantation area [2]. However, it was found that the seedlings cultivated by sexual propagation had good field performance. For example, the survival rate of afforestation, root growth potential and cumulative biomass of seedlings cultivated by nursery were better than those cultivated by cutting and other asexual propagation techniques, and they were more suitable for the harsh living environment [3,4]. Therefore, considering the differences in phenotypic traits, physiological characteristics, and environmental adaptability of seedlings with different breeding methods and the influence of environmental factors such as soil nutrient enrichment, it is very important to select the ideal propagation methods to cultivate seedlings for the growth and development of seedlings after afforestation.

The root system, the main organs of the underground part of plants absorbing nutrients, directly affects the formation of plant components and individual growth and development [5]. Under the influence of environmental factors, plants can moderately adjust the morphological structure of each component to avoid survival risks and maximize access to limited soil resources to enhance their competition and stress resistance in the community [6]. In particular, the geometric forms such as root diameter, volume, surface area and growth angle show great plasticity, which redesigns the root structure at the early stage of development [7]. Previous studies have shown that in the soil surface layer, the fine root diameter, root length density and root weight density of the *Dacryodes edulis* seedlings cultivated by seeds are larger than those of the seedlings cultivated by rooted cutting and layering. The horizontal distribution amplitude and vertical depth of fine roots increase with soil thickness, making them more competitive for underground resources than the seedlings cultivated by vegetative propagation [8]. Compared with seed propagation, the *Citrus* rootstock produced by cutting propagation is mainly composed of adventitious roots or lateral roots; the number and length of roots are larger, which is more suitable for resource acquisition [9].

Further studies showed that the root changes of *Eucalyptus pellita* seedlings cultivated by different propagation methods were obvious at the initial stage of root development after colonization. The root configuration and distribution of seedlings cultivated by the same propagation methods were similar to the increase in forest age, which was related to the genetic mechanism [10]. In addition, phenotypic traits are the basis of plant functional characteristics. Root configuration characteristics reflect the similarities and differences in morphology and arrangement of plant roots in living media under different propagation methods. In addition, they reflect their spatial location and characteristics of soil resource acquisition, which will further affect physiological and metabolic pathways such as secondary metabolites and protein synthesis in roots [11,12]. Therefore, it is very important to study the geometric configuration characteristics of seedling roots with different propagation methods at the early stage of development, especially in understanding the growth and development of roots. However, there are few studies on the seedlings of afforestation tree species, and the information on seedling roots due to differences in propagation methods is insufficient.

In subtropical China, the Chinese fir (*Cunninghamia lanceolata* (Lamb) Hook) is a high-quality, fast-growing evergreen coniferous tree. It is widely distributed and planted in southern China, the plantation area is almost 8.95 × 10^6^ ha, and the volume is 625 × 10^6^ m^3^ [13]. In a suitable environment of 30% soil moisture content, deep soil layer, heavy soil texture, and more available nitrate (N), phosphorus (P) and potassium (K) content in forest-land, Chinese fir grow quickly with high-quality wood [14,15]. The current studies on root plasticity and foraging strategies of Chinese fir have mainly focused on nutrient availability and different abiotic stresses. Such as, under the condition of low available P, Chinese fir obtained limited resources to the maximum extent through root proliferation (the increase of total root length, root surface area, root volume, and the accumulation of root dry matter mass) to improve the P utilization efficiency [16,17]. Moreover, under different planting densities, growth space and competition between adjacent roots in high-density plantations were significantly higher than those in low-density plantations, this impacting root length and volume [18]. Under severe drought conditions, the additive effect of elevated temperature affects the abundance of the arbuscular mycorrhizal fungal community and the length of extraradical hyphae, resulting in the phenotypic shaping of Chinese fir roots and the adjustment of nutrient acquisition strategies [19]. Therefore, comparative studies on the root development and configuration characteristics of Chinese fir trees with different propagation methods have been scarce. There has been little discussion on this issue, especially since the resource acquisition and life characteristics it reflects are not clear enough.

Apart from the sexual reproduction of Chinese fir seeds, asexual reproduction technology has also become an important source of afforestation materials. Different propagation methods lead to different root growth characteristics and habitat adaptation mechanisms of Chinese fir trees. Selecting the best propagation methods according to local conditions can not only improve the survival rate of afforestation but also significantly save the cost of afforestation and reproduction and reduce unnecessary waste of resources. In this paper, we focused on the difference in propagation methods using the whole nutrient solution irrigation method, the seed germination of Chinese fir cultivated by sexual reproduction, and the tissue culture and rooted cutting cultivated by asexual reproduction. We assumed: (1) there are certain differences in root architecture and spatial distribution characteristics of Chinese fir trees under different propagation methods; (2) a certain correlation is present between the change of root geometry and its growth trend in different directions. Understanding the spatial distribution and geometric morphological characteristics of the root system of Chinese fir trees with different propagation methods will help to understand its growth and development mechanism in the early initial stage and then provide a reference for the long-term scientific and sustainable management of Chinese fir plantations.

## 2. Materials and Methods

### 2.1. Plant Materials

The cultivation of different test seedlings began in early March 2020. Seeds used for seed germination were sterilized with 0.5% potassium permanganate solution, then sown at a density of 50 plants·m^−2^ on a seedbed and covered with yellow-core fine soil about 0.5 cm thick. The explants used for tissue culture were taken from semi-lignified stem segments without mechanical or pest damage. After disinfection and inoculation on a sterile operation table, adventitious bud induction and rooting culture were carried out. When the root length was about 3–5 cm, the seedlings were acclimatized. After that, the medium attached to the roots of the tissue culture seedlings was washed with water and transplanted into the soil at a density of 50 plants·m^−2^.

The scions of the rooted cutting were annual branches with terminal buds, the leaves in the form of whorls, and of length 10 cm. The cuttings were set into the soil at a density of 50 plants·m^−2^, with a spacing of 10 cm × 20 cm and a cutting depth of 3 cm. The substrate and conventional weeding, fertilization and watering management measures used during seedling breeding were the same.

In May 2021, one-year-old Chinese fir seed germination with bare roots, tissue culture and rooted cutting was taken. Due to the different propagation methods, the growth rate, development characteristics and morphological characteristics of Chinese fir trees in the three propagation methods were different. The growth traits are shown in (Table 1). Before transplanting, the seedlings were stored in the College of Forestry, Fujian Agriculture and Forestry University greenhouse on a sand-bed for 1 month; the appropriate amount of watering was used according to weather conditions.

### 2.2. Experimental Design

The sand culture experiment was performed in a greenhouse at Fujian Agriculture and Forestry University in June 2021. According to the aboveground part and root growth of the tested trees, 32 cm × 18 cm × 21 cm (upper diameter × lower diameter × high) polyethylene flowerpot was selected as the cultivation container, and a tray was arranged at the bottom of the container to avoid water loss. The dead roots of the tested seedlings were removed, and seedlings were transplanted into the cultivation container. One Chinese fir tree was planted in each container, and 9 trees were planted in each propagation method, a total of 27 trees.

To make the nutrient status of the tree growth environment consistent, washed river sand was used as the culture substrate, and a 20 kg substrate was installed in each pot. The total phosphorus content was 0.06 g·kg^−1^ (trace available phosphorus), the total potassium content was 3.23 g·kg^−1^, and the total nitrogen content was 0.28 g·kg^−1^. To meet the nutrient requirements of trees during the experiment, the whole nutrient solution was sprinkled according to the improved 1/3 Hoagland nutrient solution formula [20]. The pH of the nutrient solution was adjusted to 5.8 by 0.5 mol/L NaOH and 0.5 mol/L HCl diluted solution. The whole nutrient solution was poured once every 7 days, 100 mL for each pot and 200 mL pure water at 18:00 every day. Potted greenhouse room temperature was 18–28 °C, light 14 h·d^−1^, relative humidity >80%. The potted Chinese fir seedlings were harvested after the end of the growth period (90 days) in September 2021.

### 2.3. Harvest and Data Collection

#### 2.3.1. Measure of Root Morphological Index

To keep the spatial distribution structure of Chinese fir tree roots intact (in situ), a knife was used to gently cut the culture container along one side and then slowly separate it from the culture substrate. After stripping the culture container, the substrate was brushed slowly from top to bottom, spraying appropriate water to prevent root fracture while removing the substrate. When the main root system was exposed, the angle between it and the ground was measured with a protractor (precision 0.1°), and the main root average branch angle of the main root system was calculated. When the main roots were exposed, the included angle was measured with a protractor (precision 0.1°) to calculate the main root average branch angle.

When the intact roots were exposed, the roots’ horizontal growth amplitude and vertical extension depth were measured with a ruler (accuracy of 0.1 mm). In addition, according to the root distribution characteristics, the three-dimensional tree modeling software (SpeedTree 8.4, IDV, Lansing, MI, USA) was used to make the schematic diagram of vertical root distribution [21]. Finally, the harvested Chinese fir trees were returned to the laboratory to clean the roots with deionized water and separated from the aboveground parts. Canadian digital scanner (STD1600 Epson, Tokyo, Japan) was used to scan the root analysis system. WinRHIZO software (version 4.0 b, Quebec, QC, Canada) was used to quantitatively analyze the geometric parameters of root length, root surface area, root volume, average root diameter, root tip number and average link length.

#### 2.3.2. Measurement of Fractal Dimension of Roots

The root analysis software was used to analyze the original map after scanning. The small squares with *r* edge length and the number of small squares intercepted by roots *Nr* on the root distribution map were obtained. As the side length *r* of the small square gradually decreases, the *Nr* intercepted by the root gradually increases. After obtaining the corresponding *Nr* values at different *r* levels, log*r* and log*Nr* were used as abscissa and ordinate, respectively. The linear regression equation is:

log*Nr* = −FDlog*r* + log*K* (1)

In the Formula (1), *K* is a constant, that is *Nr*∝*r*^−FD^. The negative slope number of the regression line is FD (fractal dimension) [22].

#### 2.3.3. Measurement of the Topological Index of Roots

Fishtail branch and fork branch in the root topological structure was measured by using the proposed topological index expression of Fitter [23] and Bouma [24],

*TI* = lg*A* + lg*M* (2)

In Formula (2), *TI* is the topological index, *A* is the longest (most connected channel) internal connection number, and *M* is the total number of external connections. A closer *TI* value to one indicates that *A* and *M* are approximately equal; the root branch is less, and the root is more similar to the fishtail branch structure. A closer *TI* value to 0.5 indicates that there are relatively more external connections in the roots, and the more approximate is the branching structure of the roots.

### 2.4. Statistical Analyses

Single-factor analysis of variance (One-way ANOVA) and least significant difference test (LSD) was used to analyze the differences in root geometric morphology indexes of Chinese fir seedlings with different propagation methods. Before data analysis, the normality and homogeneity of variance were tested, and the data that did not meet the requirements were transformed into log values to meet the analysis requirements. All statistical analyses were performed using R 4.1.2 software (Robert and Ross et al. Auckland, New Zealand), and the correlation analysis was performed and visualized through the “Hmisc” package and “Corrplot” package. All the data were expressed as average values ± standard error.

## 3. Results

### 3.1. Root Plasticity under Different Propagation Methods

**Figure 1:**
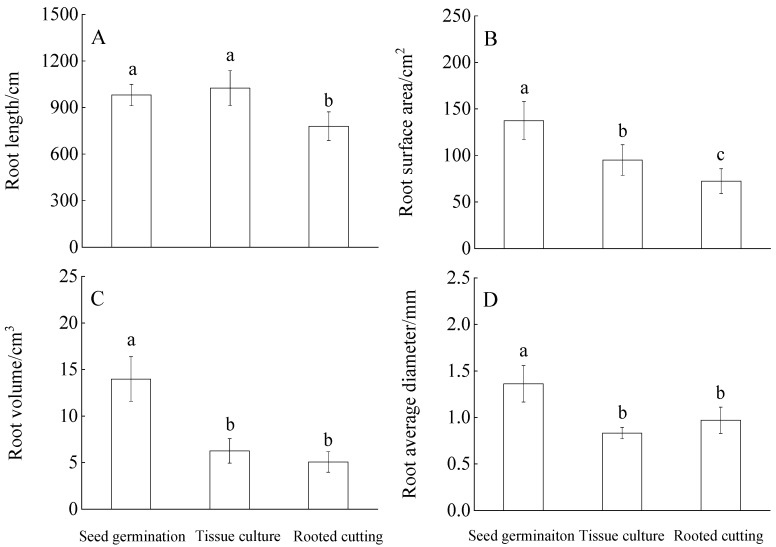
Differences in the root length (**A**), root surface area (**B**), root volume (**C**), and root-average diameter (**D**) among different propagation methods. Bars (mean ± standard error) and the different lowercase letters indicate significant differences among different propagation methods at *p* < 0.05.

The root length of seed germination and tissue culture was significantly larger than that of the rooted cuttings (*p* < 0.05), but there was no significant difference between seed germination and tissue culture (*p* > 0.05). In addition, the root surface area of seed germination was significantly larger than that of tissue culture and rooted cutting (*p* < 0.05), the rooted cutting was the smallest. The root volume and average root diameter of seed germination were significantly higher than those of tissue culture and rooted cutting (*p* < 0.05). However, there was no significant difference in root volume and average root diameter between tissue culture and rooted cutting (*p* > 0.05). On the whole, the propagation methods exhibited different effects on the root morphology of Chinese fir trees (Figure 1).

### 3.2. Root Geometric Architecture

**Figure 2:**
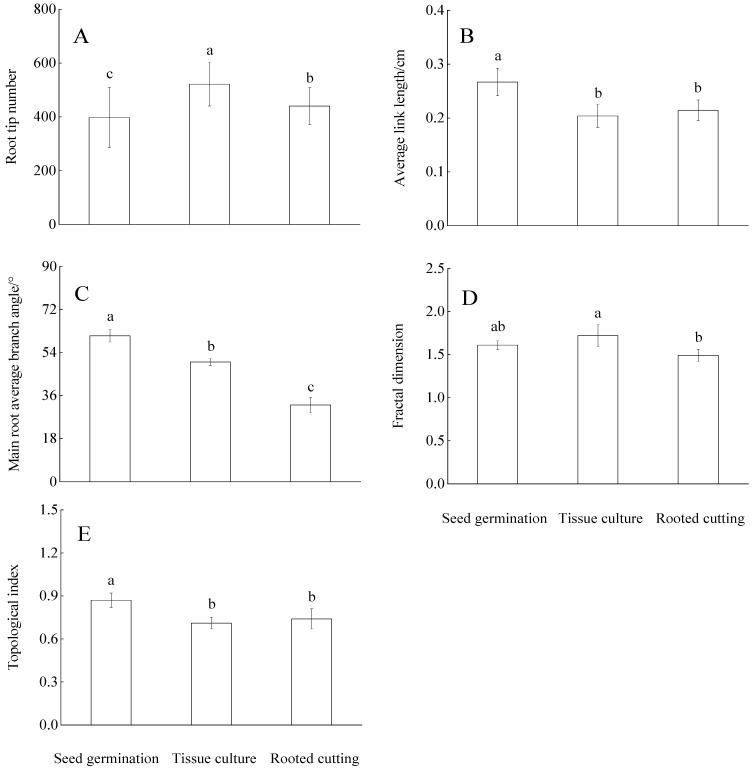
Differences in the root tip number (**A**), average link length (**B**), main root average branch angle (**C**), fractal dimension (**D**), and topological index (**E**) among different propagation methods. Bars (mean ± standard error) and the different lowercase letters indicate significant differences among different propagation methods at *p* < 0.05.

The root tip count of tissue culture was significantly higher than that of seed germination and rooted cutting (*p* < 0.05), and the root tip number of seed germination was the least. The average link length of seed germination was significantly longer than that of tissue culture and rooted cutting (*p* < 0.05), but there was no significant difference between tissue culture and rooted cutting (*p* > 0.05). The main root average branch angle of the seed germination was significantly higher than that of the tissue culture and rooted cutting (*p* < 0.05), and the rooted cutting was the smallest. The seed germination may have greater growth advantages in the vertical direction, while the tissue culture and rooted cutting grow better in the horizontal direction (Figure 2).

The fractal dimension of the root system with different propagation methods showed the order of tissue culture > seed germination > rooted cutting. Still, no significant difference was observed between seed germination and tissue culture, seed germination and rooted cutting (*p* > 0.05). According to the topological structure theory, the root TI of seed germination was close to one, and the branching pattern of the root tended to fishtail structure in space, which indicated that the root structure of seed germination was simple, the axial root was obvious, and the branching was less. The root TI of tissue culture and rooted cutting was close to 0.5, and there was no significant difference between them (*p* > 0.05). The branching pattern tended to be a forked structure, with relatively more branches, and the root branching pattern was somewhat similar (Figure 2).

### 3.3. Root Spatial Distribution

There were significant differences in the vertical distribution depth of roots under different propagation methods. The root depth of the seed germination was 17.33 ± 1.70 cm, followed by the rooted cutting, and the root depth of tissue culture was a shallow stratum (12.41 ± 1.31 cm). Still, there was no significant difference between tissue culture and the rooted cutting (*p* > 0.05). In terms of the horizontal direction, the distribution range of tissue culture (17.05 + 1.33 cm) was significantly larger than that of seed germination and rooted cutting (*p* < 0.05). There was no significant difference between seed germination and rooted cutting, and the horizontal distribution range of seed germination was the narrowest (Table 2).

In addition, the root width ratio to root germination depth was the lowest (0.72 ± 0.09). The ratio of root width to root depth in tissue culture was the highest (1.59 ± 0.25). The difference between rooted cutting and seed germination was significant (*p* < 0.05), but the difference between seed germination and tissue culture was not significant (*p* > 0.05). It can be seen that the root depth of the seed germination was the deepest. Still, the horizontal growth range was the narrowest, and the root growth trend in the vertical direction was strong. The root of the tissue culture had a greater growth advantage in the horizontal direction. The root growth distribution of the rooted cutting was between the seed germination and the tissue culture in both vertical and horizontal directions. It had more similar growth characteristics to tissue culture (Table 2).

**Figure 3:**
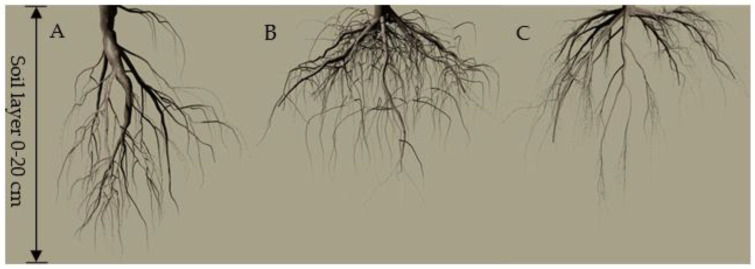
The root vertical distribution schematic diagram originated from seed germination (**A**), tissue culture (**B**), and rooted cutting (**C**).

There was a significant difference in the spatial distribution characteristics of the Chinese fir root system under different propagation methods. In the vertical direction, the root system of seed germination had obvious and serpentine axial root, with lateral roots growing from the axial root and smaller lateral roots from the lower level, forming a root structure system inclined to vertical growth. The root distribution of tissue culture and rooted cutting was similar. There was no obvious axial root in the vertical direction, but multiple lateral roots mainly radiated along the horizontal direction from the independent dry base (Figure 3).

### 3.4. Correlation between Spatial Distribution and Geometric Morphology

**Figure 4:**
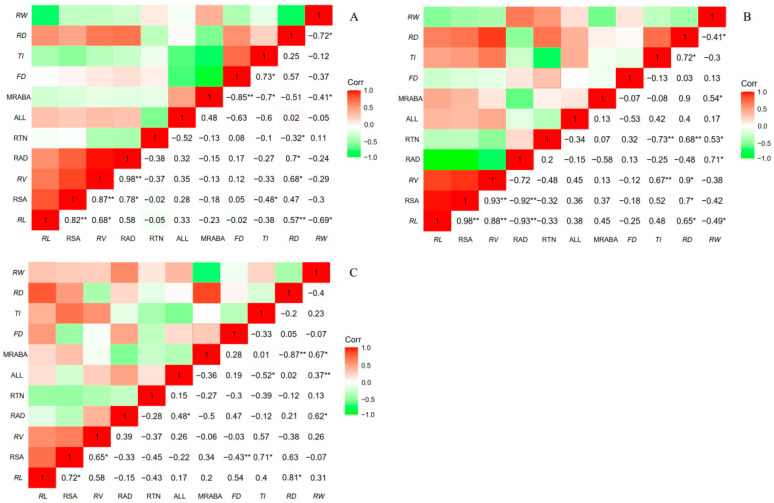
Correlation analysis between spatial distribution and geometric morphology among different propagation methods of seed germination (**A**), tissue culture (**B**), and rooted cutting (**C**). * means significant difference (*p* < 0. 05); ** means extreme significant difference (*p* < 0.01). RL = root length; RSA = root surface; RV = root volume; RAD = root average diameter; RTN = root tip number; ALL = average link length; MRABA = main root average branch angle; FD = fractal dimension; TI = topological index; RD = root depth; RW = root width.

The root depth of seed germination, tissue culture, and root cutting was significantly positively correlated with root length, root volume, and root-average diameter; however, it was significantly negatively correlated with root tip count. Root depth of tissue culture was positively correlated with root surface area, topological index, and root tip count. The root depth of the rooted cutting negatively correlated with the main root average branch angle (Figure 4).

The root width of seed germination was negatively correlated with root length, root average branch angle, and depth, as was the tissue culture. The root width of tissue culture and rooted cutting were positively correlated with the root average diameter and the main root average branch angle. The root width of tissue culture was also significantly positively correlated with the root tip count. The root width of the rooted cutting was positively correlated with the fractal dimension.

## 4. Discussion

Changes in the morphological structure during root development are mainly affected by genetic characteristics and external environmental factors. These factors cause changes in signal substances such as internal hormones, which eventually influence root growth plasticity and flexibility. However, when the living environment is the same, the root morphology changes depending on the tree species genetic characteristics [25]. Our study showed differences in root morphology of Chinese fir trees due to different propagation methods under the same environmental conditions, and nutrients provided by the soil did not restrict root growth. Root morphological characteristics reflect the plants’ long-term evolutionary ability to acquire and utilize resources [26]. Usually it can be said, the larger the root diameter, the less developed the cortex and the weaker the root absorption capacity. However, a larger root diameter induces stronger transport capacity.

Root length can characterize its expansion ability in soil space, and root surface area and volume reflect the contact area between root and soil [27]. In this study, the root average diameter of Chinese fir trees showed that the seed germination methods had larger values than the tissue culture and the rooted cutting. The tissue culture’s root length and surface area were significantly larger than that of the rooted cutting. Still, the two groups showed no significant differences in root volume and average diameter (Figure 1). The roots of Chinese fir trees with different propagation methods can be found to have different soil resource absorption characteristics. These characteristics are related to the dominant role of the genetic mechanism determined by genes in the regulation of root development at the early stage of the root development [28]. It is widely accepted that morphological differences imply some degree of difference in root geometry, and our study also confirms this view.

This study found different degrees of differences in root geometry of Chinese fir trees among propagation methods (Figure 2). Van de Bom et al. [29] pointed out that root configuration can reflect the input and tradeoff strategy of metabolic cost when plants capture soil resources, establishing an ideal root configuration beneficial to soil resource absorption through root growth angle and root branching. In this study, the average link length and main root branch angle of the seed germination method were significantly higher than that of tissue culture and rooted cutting. It was found that the smaller the angle between root and ground, the wider the horizontal growth space. In the same growth medium, the horizontal expansion range of seed germination is smaller than that of tissue culture and rooted cutting, which may also be why the root tip count of seed germination is less than that of tissue culture and rooted cutting. According to the optimal allocation theory, when plants reduce the energy input of a certain component, the components beneficial to resource acquisition will have the opportunity to obtain more energy, and the seed germination will use more C for root length construction [30].

In this study, seed germination roots had obvious axial roots in the vertical direction and deeper roots than tissue culture and rooted cutting. Tissue culture and rooted cutting did not have the obvious axial and vertical roots, but lateral roots had developed (Figure 3). Combined with Table 2, it can be seen that the root system of seed germination constructs a “compact” vertical root configuration in the vertical direction. The root system of tissue culture and rooted cutting tends to construct a “diffusion” horizontal reticular root configuration in the horizontal direction, which further indicates differences in root configuration of Chinese fir seedlings under different propagation methods. Different life strategies are shown under the same habitat, which confirms our hypothesis that propagation methods affect the root configuration of Chinese fir.

To further understand the root architecture characteristics and soil resource foraging characteristics of Chinese fir seedlings with different propagation methods, we further quantified the root phenotype by fractal dimension and a topological index. We found differences in the fractal dimension of the root system of Chinese fir seedlings under different reproduction methods. Walk et al. [22] considered that the fractal dimension represented the complexity of the root structure. The larger the fractal dimension, the more complex was the root structure, and with a tendency to grow horizontally. Therefore, the root structure of tissue culture is more complex than that of rooted cutting and seed germination, mainly caused by the characteristics of asexual propagation methods. Many adventitious roots are generated at the base of the trunk and gradually develop to form developed lateral roots to construct a complex horizontal root network system. However, these “growth characteristics” controlled by genetic mechanisms may show different patterns of change under stress, such as temperature, water, and nutrients [31,32].

One possible reason that the fractal dimension of seed germination was greater than that of rooted cutting was that this study provided sufficient water and nutrients for the seedling’s growth. The growth trend of seed germination roots in the vertical direction is greater than that of rooted cutting in the horizontal direction. The longitudinal extension ability of the axial root is greater than that of the lateral root of rooted cutting. The final vertical root network system is more complex than a horizontal root network system constructed by rooted cutting [33]. According to the topological index, the roots of seed germination showed a fishtail-shaped branching structure. In contrast, the roots of tissue culture and rooted cutting belonged to a fork-shaped branching structure.

The C construction cost of root fishtail branching mode is generally believed to be high, but the competition within the root system is small. It is abler to adapt to drought and barren nutrient environments, the C construction cost of the root system approaching the forked branching mode was low, and the internal competition of the root system was more intense. Still, the root system grew rapidly [34,35]. It further indicated that the seed germination had the characteristics of reducing the number of lateral roots and horizontal expansion to invest more C costs into the longitudinal axial root growth; tissue culture and rooted cutting occupy more space, limited resources, and invest more C costs for lateral root growth by not having vertical axial root, forming a complex horizontal network structure of roots. The comprehensive growth trend in the vertical and horizontal directions creates the spatial distribution structure of the root system of Chinese fir seedlings with different propagation methods. This growth characteristic further reflects that the root system of Chinese fir seedlings with different propagation methods tends to be different regarding resource acquisition and structural method construction. Still, they all construct a root system conducive to enhancing their competitiveness towards the goal of the most efficient utilization of resources to expand and adapt to different growth spaces and to form their own root development characteristics to cope with the living environment [32,36].

Previous studies have shown that narrow and deep root configurations can capture more spatial resources. In contrast, wide and shallow root configurations can expand the occupation and utilization of shallow soil space, and shallow roots can better absorb nutrients concentrated on the soil surface with low mobility, such as phosphorus [29]. Therefore, one of the life strategies of Chinese fir seed germination roots of steep and deep roots is to find more space resources. Longitudinal growth of the main roots enables it to have the basic conditions for exploring and absorbing deep soil nutrients. It can obtain nitrate and water in deeper soil layers in harsh environments. Still, at the same time, it may reduce the acquisition of nutrients such as phosphorus and potassium from shallow topsoil. The surface soil foraging characteristics of tissue culture and rooted cutting occupy limited space resources to the maximum [37]. However, it was found in previous studies that Chinese fir seed germination only had obvious axial roots in the first few years of the seedling stage. With the increase of forest age, lateral roots gradually developed, and the axial roots were not obvious [18], as most plants’ root depth and width will increase by a certain proportion [38,39]. After afforestation, with the increase of stand age, it remains to be further studied whether the seed germination has a stronger resource predation ability in the seedling stage compared with tissue culture and rooted cutting in the barren environment.

The changes of root and canopy phenotypic traits affects its spatial distribution pattern, and there is a correlation between root configuration adjustment and its growth trend in different directions [40,41]. The correlation analysis between spatial root distribution and geometric morphology of Chinese fir seedlings under different propagation methods showed a strong positive correlation between root depth and root length of Chinese fir seedlings under different propagation methods. These results indicated that the expansion of the root system of Chinese fir seedlings in different propagation methods could be achieved by increasing the root length by investing more carbon cost. There is a negative correlation between root width and root depth of seed germination, tissue culture, and rooted cutting, indicating a certain tradeoff between root depth and root width. This tradeoff may play an important role in the metabolic cost of plants acquiring resources in nutrient heterogeneous patches. When the roots reduce the horizontal growth range and invest in vertical growth, they can capture deeper soil resources, and the obtained resources promote the roots’ horizontal growth [34]. It was also further shown that when the benefits of plant investment on resource acquisition are negligible, more carbon costs will be invested in resource capture-friendly components, a relationship consistent with the optimal allocation theory and the balanced growth hypothesis of the plant resource allocation pattern [30,42]. In this process, the root volume, root surface area, branch angle, root depth, and root width showed different correlations, reflecting the root morphological plasticity changes.

## 5. Conclusions

In this study, we found that the root development characteristics of Chinese fir trees cultivated by sexual and asexual propagation techniques differed in the initial stage. There were differences in root morphology, spatial distribution, and resource acquisition characteristics of Chinese fir under seed germination, tissue culture, and rooted cutting methods. The root system of Chinese fir seed germination showed a fishtail branch structure, tending to construct a root system in the vertical direction. The root system of tissue culture and rooted cutting showed a forked branching structure, mainly caused by the growth characteristics of seedlings cultivated by asexual propagation. Due to the differences in resource capture characteristics of different propagation methods of Chinese fir trees, the horizontal occupancy space of the root system of propagation is less than that of the tissue culture and rooted cutting. However, it makes up for the limitation of resource acquisition in the horizontal direction by constructing an axial root to seek deeper soil space resources. Due to the absence of vertical axial roots, tissue culture and rooted cutting were allocated limited resources to lateral roots to promote their growth and reproduction to perform anchoring and foraging functions, resulting in a highly complex root network. Through correlation analysis, it was found that the root system of Chinese fir trees with different propagation methods showed a tradeoff between “seeking” and “occupying” spatial resource growth in the vertical and horizontal directions, which was closely related to the change of root geometry. This result was conducive to selecting the most favorable propagation methods of Chinese fir trees according to site conditions and afforestation costs in production. However, the change in the root system will further affect the morphological construction and physiological characteristics of aboveground plants; therefore, the influence of root configuration differences on the growth, nutrient absorption, and utilization characteristics of the whole plant needs further study.

## Figures and Tables

**Table 1:** Height and root collar diameter before transplanting of Chinese fir trees.

Propagation Methods	Height/cm	Diameter at the Root Collar/cm
Seed germination	25.92 ± 2.84b	0.40 ± 0.05b
Tissue culture	29.20 ± 3.73a	0.49 ± 0.08a
Rooted cutting	23.10 ± 2.66b	0.38 ± 0.06b
*p*-value	<0.001	<0.001

Note: Different lowercase letters indicate significant difference between propagation methods at *p* < 0.05.

**Table 2:** Root spatial distribution traits. The same lowercase letters are not significantly different at the 5% probability level, while different lowercase letters indicate significant differences between the propagation methods.

Propagation Methods	Root Depth/cm	Root Width/cm	Root Width/Root Depth
Seed germination	17.33 ± 1.70a	10.87 ± 0.80b	0.72 ± 0.09b
Tissue culture	12.41 ± 1.31b	17.05 ± 1.33a	1.59 ± 0.25a
Rooted cutting	15.20 ± 1.57ab	13.57 ± 0.82b	1.06 ± 0.16b

## Data Availability

All the data is presented in the paper.

## Abbreviations

RL = root length; RSA = root surface; RV = root volume; RAD = root average diameter; RTN = root tip number; ALL = average link length; MRABA = main root average branch angle; FD = fractal dimension; TI = topological index; RD = root depth; RW = root width.
